# Scrotal Hirudiniasis: The Mysterious Migration of a Leech Into the Scrotum: A Case Report

**DOI:** 10.1002/ccr3.72544

**Published:** 2026-04-12

**Authors:** Pranto Chakroborty, Md. Abdul Quadir

**Affiliations:** ^1^ FCPS Part ‐ II Trainee (Neurosurgery) Sylhet MAG Osmani Medical College Hospital Sylhet Bangladesh; ^2^ Associate Professor, Department of Surgery Sylhet MAG Osmani Medical College Hospital Sylhet Bangladesh

**Keywords:** acute scrotum, case report, hirudiniasis, leech, parasitic infestation

## Abstract

Hirudiniasis, or leech infestation, is a rare parasitic condition typically involving external skin or mucosal surfaces. We report a case of scrotal hirudiniasis, an 11 cm long leech discovered within the scrotal sac of a 15‐year‐old male. He initially developed hematuria due to periurethral leech attachment, which resolved following catheterization and bladder irrigation. A week later, he presented with acute scrotal pain and swelling. Ultrasound revealed a tubular, mobile structure near the left vas deferens. Surgical exploration identified an 11 cm leech within the tunica vaginalis, without any external entry wound or urethra‐cutaneous fistula. Two plausible routes of entry are suggested: retrograde movement via the vas deferens or migration through perineal fascial planes following urethral breach. This case underscores the need to consider unusual parasitic causes in patients presenting with acute scrotum in endemic regions.

## Key Clinical Message

Scrotal hirudiniasis is an exceptionally rare presentation of leech infestation. Clinicians should consider parasitic causes in acute scrotal swelling, particularly in patients with freshwater exposure, as early surgical exploration is both diagnostic and curative.

## 1 Introduction

Leeches are hematophagous annelid parasites (class Hirudinea) characterized by two muscular suckers and a protrusible proboscis [1, 2]. Parasitic infestation by leeches is a rare occurrence, which is medically termed as Hirudiniasis. Leeches typically inhabit freshwater in tropical and subtropical regions and attach to hosts by biting exposed skin or entering body orifices [3, 4]. Although only a few of the > 600 leech species bite humans, cases have been reported in people who drink or bathe in infested streams and ponds [3, 4]. Their human infestations are more commonly endemic in warm, tropical regions (e.g., the Mediterranean, Africa, and Asia), although may occur worldwide [2, 5]. Leeches secrete an anesthetic and anticoagulant (hirudin), often making their bites painless and prone to prolonged bleeding [3, 4]. Typically, external skin bleeding or mucosal blood loss (epistaxis, hemoptysis, hematemesis) is seen when leeches attach externally. While leech bites usually occur externally, a few cases of internal infestations have also been reported. Internal hirudiniasis most commonly involve mucosal surfaces of the upper aerodigestive tract. Iynen et al. described a nasopharyngeal leech presenting with epistaxis and hemoptysis [2], Agarwal and Gupta similarly reported nasopharyngeal infestation causing recurrent epistaxis [6]. Rai et al. further highlighted that leech infestation may remain unnoticed and present as recurrent nasal bleeding in endemic regions [4]. In addition, Askari and Eshaghian reported otorrhagia due to leech attachment in the external auditory canal, demonstrating the parasite's ability to invade various orifices [3].

Genitourinary hirudiniasis is especially uncommon. Most reported cases involve children from endemic regions presenting with hematuria or urinary obstruction. Reported genitourinary hirudiniasis cases typically present with hematuria, dysuria, or urinary retention [1, 7]. For example, Datta et al. described a 16‐year‐old boy, of whom leeches in bladder caused gross hematuria and urinary retention [7]. Even vaginal or vulvar hirudiniasis has also been reported, which led to severe bleeding or anemia in young girls and older women. However, to our knowledge, no prior case has been reported of a leech infesting the scrotal sac. We report a unique case of scrotal hirudiniasis (a live leech in the scrotum) and explore possible anatomic routes of migration for this parasite.

## 2 Case History/Examination

A 15‐year‐old previously healthy adolescent male presented after bathing in a pond where several leeches attached to his lower limbs and external genitalia. The attached leeches were manually removed at a local healthcare facility. Shortly afterward, he developed gross hematuria and underwent urethral catheterization with bladder irrigation. The catheter was maintained for approximately 24 h, after which the hematuria resolved spontaneously. He had no prior history of urologic disease, trauma, bleeding disorders, or chronic illness, and there was no relevant family history. He was not taking any medications and had no known allergies.

However, a week after recovery, he developed sudden onset painful swelling of the scrotum, with no relevant associated history of trauma, dysuria, or fever. Physical examination revealed a tender, swollen scrotum with intact overlying skin and no visible bite mark or puncture wound.

## 3 Differential Diagnosis, Investigations and Treatment

Based on the clinical presentation of acute scrotal pain and swelling, initial consideration was given to common causes of acute scrotum, particularly epididymo‐orchitis and testicular torsion. However, the absence of fever and urinary symptoms did not strongly support these differentials.

Laboratory investigations, including hemoglobin, total leukocyte count, platelet count, renal function tests, and coagulation profile, were within institutional reference ranges, and urinalysis was also normal. Scrotal ultrasonography demonstrated a tubular, mobile echogenic structure in proximity to the left vas deferens. These findings were atypical for common inflammatory or vascular causes and raised suspicion of an unusual intraluminal pathology.

In the context of recent freshwater exposure and prior leech attachment, the imaging findings led to a strong suspicion of parasitic infestation or an unusual foreign body. Based on these findings and the patient's acute symptoms, surgical exploration was planned as both a diagnostic and therapeutic intervention.

**FIGURE 1:**
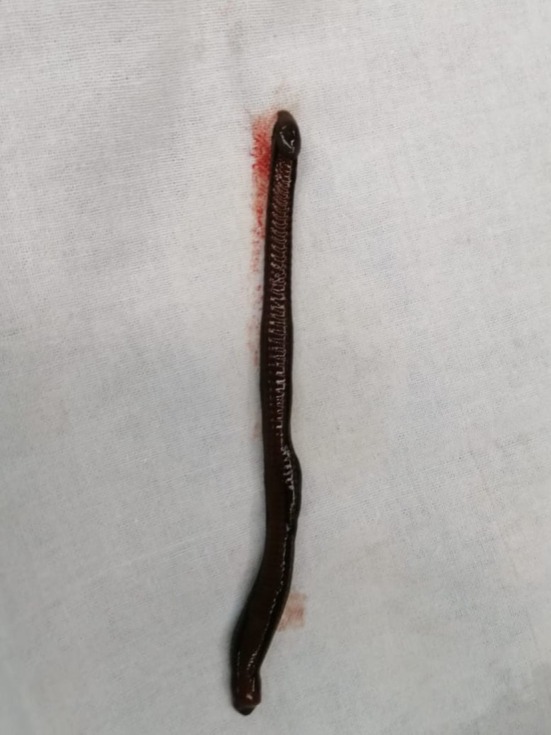
11 cm long leech removed from scrotum.

Under sub‐arachnoid block, a left para‐raphean incision was made and the layers were dissected sequentially to open the tunica vaginalis. Approximately 50 mL of dark serosanguinous fluid was drained, and a live leech measuring about 11 cm was identified and removed intact using DeBakey forceps (Figure 1, Video 1). The testis, epididymis, and spermatic cord appeared structurally normal, with no evidence of hydrocele, hernia, or urethrocutaneous communication. The cavity was irrigated with saline, hemostasis achieved, and the layers closed anatomically.

**VIDEO 1:** Peroperative removal of a live leech from the tunica vaginalis during surgical exploration of the scrotum. Video content can be viewed at https://onlinelibrary.wiley.com/doi/10.1002/ccr3.72544.

## 4 Conclusion and Results (Outcome and Follow‐Up)

Postoperatively, the patient was managed with oral antibiotics and analgesics, along with scrotal support and daily wound dressing. The postoperative course was uneventful, with no evidence of infection, hematoma, or recurrence of swelling. Pain gradually subsided, and the surgical wound showed satisfactory healing. Sutures were removed on postoperative day 7. On follow‐up, the patient remained asymptomatic with a normal scrotal examination and no evidence of recurrence or complications.

## 5 Discussion

Internal leech infestations involving the genitourinary tract have been documented almost exclusively in endemic rural areas, typically in children who swim or bathe in leech‐infested water [6]. In such cases, leeches usually enter via the urethra and lodge in the bladder or lower urinary tract, causing painless bleeding [6]. Most reported cases present with hematuria, bladder spasms, urinary retention, or visualization of a leech at the urethral meatus [1, 7]. For example, Datta et al. described a teenage boy with sudden lower abdominal pain and profuse hematuria; imaging and cystoscopy revealed an 8.5 cm leech in the bladder wall [7]. In many urinary‐leech cases, the parasite is often extracted cystoscopically [6]. Vaginal or vulvar infestation has also been reported in prepubertal girls, often with unexplained vaginal bleeding [1, 8]. Although uncommon, nasal and oropharyngeal leech infestations are also reported, which most often present with epistaxis or throat bleeding [4, 6]. However, to our knowledge, infestation of the scrotal sac by a leech has not previously been reported in the literature.

In our patient, a large (11 cm) live leech was discovered within the tunica vaginalis during exploration for a spontaneous hematocele. No visible external wound or identifiable urethral fistula was seen. This unusual finding raises an important question regarding the route of entry. Based on the clinical findings and intraoperative exploration, we propose two possible pathways, both rooted in anatomical feasibility and supported indirectly by prior literature.

### 5.1 Intraluminal Retrograde Migration via Vas Deferens

A possibility is that the leech traveled retrograde from the urethra through the ejaculatory ducts into the vas deferens, reaching the epididymal end. From there, it might have breached the wall of the vas deferens to enter the scrotum. It is reported that inflammatory conditions of the vas deferens (vasitis) are attributed to pathogens moving backward from the urethra into the vas. For example, Chen et al. (2019) stated that acute infective vasitis is thought to be due to the retrograded spread of common urinary pathogens into the vas deferens [9]. This demonstrates that organisms (or fluid) can travel through the ejaculatory ducts to reach the vas, supporting the possibility of a leech similarly migrating along this lumenal path. Also, DiMare et al. (2025) described that pediatric epididymitis can be caused by reflux of urine into the ejaculatory ducts as a common mechanism [10]. This indicates that material in the urethra can be driven into the ejaculatory ducts and vas deferens, again implying an anatomical route from the urethra to the scrotal contents. And, Leeches of the family Arhynchobdellida (with strong jaws) are known to bite through tough tissues, and their secreted anesthetic (hirudin‐related) makes these bites painless [1]. Thus, a silent mucosal bite in the vas could allow retrograde entry without obvious hemorrhage at the time of ingestion. Applying this to our case, we suggest a similar route might have been exploited by the parasite. While there is no direct precedent for a leech using this path, the anatomical continuity provides a theoretical basis.

### 5.2 Migration Along Perineal Fascial Planes

A minor mucosal breach in the bulbar urethra might have allowed the leech to exit into the surrounding soft tissue. From there, it could have tracked along the fascial planes through Colles' fascia and into the dartos fascia of the scrotum. Anatomical texts note that “in males, Colles' fascia continues into the scrotum, where it encloses the dartos muscle” [11]. The spread of infection or foreign bodies along Colles' and dartos fascia to the scrotum is well recognized in conditions such as Fournier's gangrene and spermatic cord infections [12]. Any fluid, infection, or organism escaping from the urethra or perineal tissues can easily track into the scrotum, as these fascial layers are continuous. Supporting this, Medscape's anatomical review explains that infections of the urethra or superficial perineum can “drain into the superficial perineal space and can extend into the scrotum” [13]. Similarly, in cases of anterior urethral injury with disruption of Buck's fascia, scrotal enlargement commonly occurs because extravasated fluid is confined only by Colles' fascia [14].

There is also a published case that provides direct evidence of communication from the urethra to the scrotum. Djatisoesanto et al. (2021) [15] describe a paraplegic patient who developed a urethroscrotal fistula and scrotal abscess. Retrograde urethrogram in that case showed contrast material spreading into the scrotum from a urethral defect. Extrapolating from their findings, it is reasonable to consider that a flexible organism such as a leech could follow similar planes.

It's also important to mention another possible pathway of leech entry to scrotum. Leeches possess the ability to attach and penetrate moist, vascular skin—especially in humid environments. This Leech might have directly penetrated scrotal skin and entered scrotal sac. However, in our case, no bite marks, puncture wounds, or dermal breaches were observed clinically or during surgery. Hence, while transdermal entry remains a theoretical route, it is considered less likely than transurethral migration in this case.

Both routes are speculative due to lack of direct evidence. However, several aspects support their plausibility. Leeches have been recovered from very narrow ducts in humans (e.g., ureters, urethra) and are known to survive extended periods attached internally [1, 5]. Their bites are characteristically painless and leave a “Y‐shaped” wound that bleeds easily [1], so one might not recall any trauma or pain. Advanced imaging might have detected a small tract, but such detailed urogenital imaging was not performed due to resource limitations in our setting.

Previously reported internal hirudiniasis cases were managed primarily through endoscopic or direct extraction. In contrast, our case required surgical exploration because the parasite was located within the tunica vaginalis and could not be accessed by minimally invasive means. This distinction highlights how anatomical location determines both presentation and treatment approach. In endemic settings, patients presenting with hematuria, unexplained bleeding, or acute scrotal swelling after freshwater exposure should be evaluated for parasitic infestation. Initial assessment should include detailed exposure history and physical examination. Ultrasound serves as a first‐line imaging modality which plays a crucial role in differentiating parasitic infestations from testicular torsion, epididymo‐orchitis, hydrocele, or hematoma. Real‐time imaging can reveal mobile tubular structures suggestive of a parasite and guide timely surgical decision‐making. Endoscopic evaluation may be required when urinary tract involvement is suspected. Surgical exploration is indicated when imaging is inconclusive or complications such as hematocele or severe pain occur.

## 6 Strengths and Limitations

This is a rare case of scrotal hirudiniasis, providing new insights into an unreported manifestation of leech infestation. Detailed clinical and intraoperative findings were documented, allowing formulation of plausible anatomical routes of parasite migration.

However, as this is a single reported case, the proposed causal mechanisms and anatomical pathways remain speculative. The original ultrasonography file is unretrievable because the equipment used lacked digital storage or export capability, which is common in many rural healthcare facilities in developing countries. Also, advanced imaging such as a retrograde urethrogram could have offered additional insights into the parasite's entry route, but this was not feasible in our resource‐limited setting. Despite these limitations, the sonographic findings were contemporaneously documented in the patient's records and verified by the attending clinicians. Additionally, due to the rarity of the condition, comparative data or precedent literature are extremely limited, restricting broader generalization of the proposed hypotheses.

## 7 Conclusion

Leech infestation in the scrotum is an exceptionally rare occurrence. Our case underscores that leeches can invade unusual body sites and cause significant complications. Here, two plausible entry mechanisms are proposed: transurethral migration via the vas deferens or via perineal fascial planes. Clinicians in endemic regions should consider rare parasitic causes of acute scrotal swelling, particularly in patients with freshwater exposure. Surgical exploration remains essential for both diagnosis and treatment in such unusual presentations.

## Author Contributions

**Pranto Chakroborty:** conceptualization, data curation, formal analysis, investigation, methodology, writing – original draft, writing – review and editing. **Md. Abdul Quadir:** data curation, validation, writing – review and editing.

## Funding

The authors have nothing to report.

## Disclosure

Permission to Reproduce Material From Other Sources: Not applicable; no third‐party copyrighted material was reproduced.

## Ethics Statement

Ethical approval was not required for this single‐patient case report as per institutional policy; all procedures were conducted in accordance with the ethical standards of the Helsinki Declaration.

## Consent

Written informed consent was obtained from the patient's guardian for publication of this case report and accompanying images/video.

## Conflicts of Interest

The authors declare no conflicts of interest.

## Data Availability

All data supporting this case report can be made available on reasonable request.
